# Foliar application of procyanidins enhanced the biosynthesis of 2-acetyl-1-pyrroline in aromatic rice (*Oryza sativa* L.)

**DOI:** 10.1186/s12870-022-03775-7

**Published:** 2022-07-29

**Authors:** Haowen Luo, Meiyang Duan, Pipeng Xing, Huifang Xie, Xiangru Tang

**Affiliations:** 1 State Key Laboratory for Conservation and Utilization of Subtropical Agro-Bioresources, College of Agriculture, South China Agricultural University, Guangzhou, 510642 China; 2 Scientific Observing and Experimental Station of Crop Cultivation in South China, Ministry of Agriculture and Rural Affairs, Guangzhou, 510642 China; 3 Guangzhou Key Laboratory for Science and Technology of Aromatic Rice, Guangzhou, 510642 China

**Keywords:** 2-acetyl-pyrroline, Aromatic rice, Procyanidins, Gene expression, Enzyme

## Abstract

**Background:**

Procyanidins is a polyphenolic compound with multiple properties. However, the application of exogenous procyanidins in crops has not been reported. Aromatic rice is a high-quality rice with a special aroma and popular with consumers. The 2-acetyl-1-pyrroline (2-AP) is a key compound of aromatic rice aroma. In the current study, aromatic rice plants were sprayed with procyanidins solutions at 0.25 (Pr0.25), 0.50 (Pr0.50), 1.00 (Pr1.00), 2.00 (Pr2.00) g L^−1^, respectively and treatment sprayed with distilled water was taken as control (CK). The effects of exogenous procyanidins on growth and 2-AP biosynthesis of aromatic rice plants were explored.

**Results:**

Compared with CK, Pr1.00 and Pr2.00 treatments significantly increased 2-AP content by 16.67% and 37.68%, respectively. Higher proline, 1-pyrroline-5-carboxylic acid (P5C), 1-pyrroline, methylglyoxal contents, and lower γ- aminobutyric acid (GABA) content were recorded in Pr1.00 and Pr2.00 treatments than CK. Compared with CK, Pr1.00 and Pr2.00 treatments significantly improved the activities of P5CS and OAT and diminished the activity of BADH. Furthermore, compared with CK, Pr1.00 and Pr2.00 treatments significantly up-regulated the transcript levels of *P5CS2*, *P5CR*, *OAT*, *DAO4* and down-regulated the transcript levels of *BADH2*. Exogenous procyanidins had no substantial effects on plant height, stem diameter, fresh weight, and dry weight of aromatic rice plants.

**Conclusions:**

In conclusion, our findings reported the increment of 2-AP content in aromatic rice under exogenous procyanidins. Our results indicated that the application of exogenous procyanidins enhanced 2-AP biosynthesis by improving proline biosynthesis and inhibiting GABA formation.

## Background

Aromatic rice is a special rice type famous for its unique aroma and good quality [1]. Normally, the price of aromatic rice is much higher than that of non-aromatic rice, and the economic return of aromatic is also lucrative to farmers [2]. In recent years, more and more scientists have made attempts to enhance the productivity or quality of aromatic rice to improve its economic performance [3, 4]. 2-acetyl-1-pyrroline (2-AP) is the key component of the special aroma of aromatic rice cultivars. The biosynthetic mechanism of 2-AP in aromatic rice is very complicated. In the past two decades, many scientists have tried to find out how 2-AP is produced and those related factors. In 2002, Yoshihashi et al. [5] found that the nitrogen in 2-AP came from proline, and supplements of proline, ornithine, and glutamic acid increased 2-AP content in aromatic rice plants. In 2008, the presence of a dominant *BADH2* allele encoding betaine aldehyde dehydrogenase (BADH) was found to inhibit the formation of 2-AP in aromatic rice [6]. In 2015, the study by Kailkavoosi et al. [7] showed that the expression of genes related to 1-Pyrroline-5-Carboxylate Synthetase (P5CS) has substantial influences on 2-AP biosynthesis in aromatic rice. In 2016, Poonlaphydecha et al. [8] demonstrated that 1-pyrroline is a limiting substrate in 2-AP formation.

During the growing period of aromatic rice plants, the 2-AP biosynthesis is influenced by many environmental factors. For example, increased soil salinity significantly increased 2-AP content in aromatic rice [9]. The relative lower temperature during the grain-filling stage enhanced the biosynthesis of 2-AP [10]. Relative lower soil moisture was also found to substantially increase 2-AP content by up-regulating the expression of the gene *DAO1* [11]. Moreover, an earlier study found that zinc and lanthanum increased 2-AP content in aromatic rice [12]. Previous studies showed that foliar application of zinc and selenium promoted the 2-AP content [13, 14]. Furthermore, some scientists invented some cultivation measures or products, such as a special fertilizer, to increase 2-AP content based on these responses of 2-AP to different factors [15]. The study by Mo et al. [16] demonstrated that cultivation with mild drought and extra nitrogen input improved both grain yield and 2-AP content of aromatic rice.

Procyanidins is a pigment component in plants, which widely exists in various plants. Structurally, procyanidins is composed of different amounts of catechins or epicatechins [17]. Previous studies indicated that procyanidins is a well-known radical-scavenging component and useful in treating chronic metabolic diseases such as cancer, diabetes, and cardiovascular disease [18, 19]. An earlier study showed that rat pheochromocytoma cells exposed to procyanidins exhibited higher activities of antioxidant enzymes, including glutathione peroxidase, superoxide dismutase, and catalase [20]. The study by Fang et al. [21] also indicated that adding procyanidins to the diet could promote antioxidant enzymes and thus suppress weaning stress in weanling piglets. Normally, the procyanidins content is low in non-colored rice varieties, and at present, there is no research have shown whether there is a link between procyanidins and 2-AP in aromatic rice. The application of exogenous procyanidins in plants, especially in crop production, has not been reported either.

For now, whether exogenous procyanidins would influence 2-AP biosynthesis in aromatic rice plants is unknown. Hence, we conducted a pot experiment to explore the effects of foliar application of procyanidins on 2-AP content and its related mechanism in aromatic rice. The finding of the present study will provide new sight of procyanidins and reveal the potential of procyanidins as a plant growth regulator.

## Results

### Effects of exogenous procyanidins on 2-AP content

**Fig. 1:**
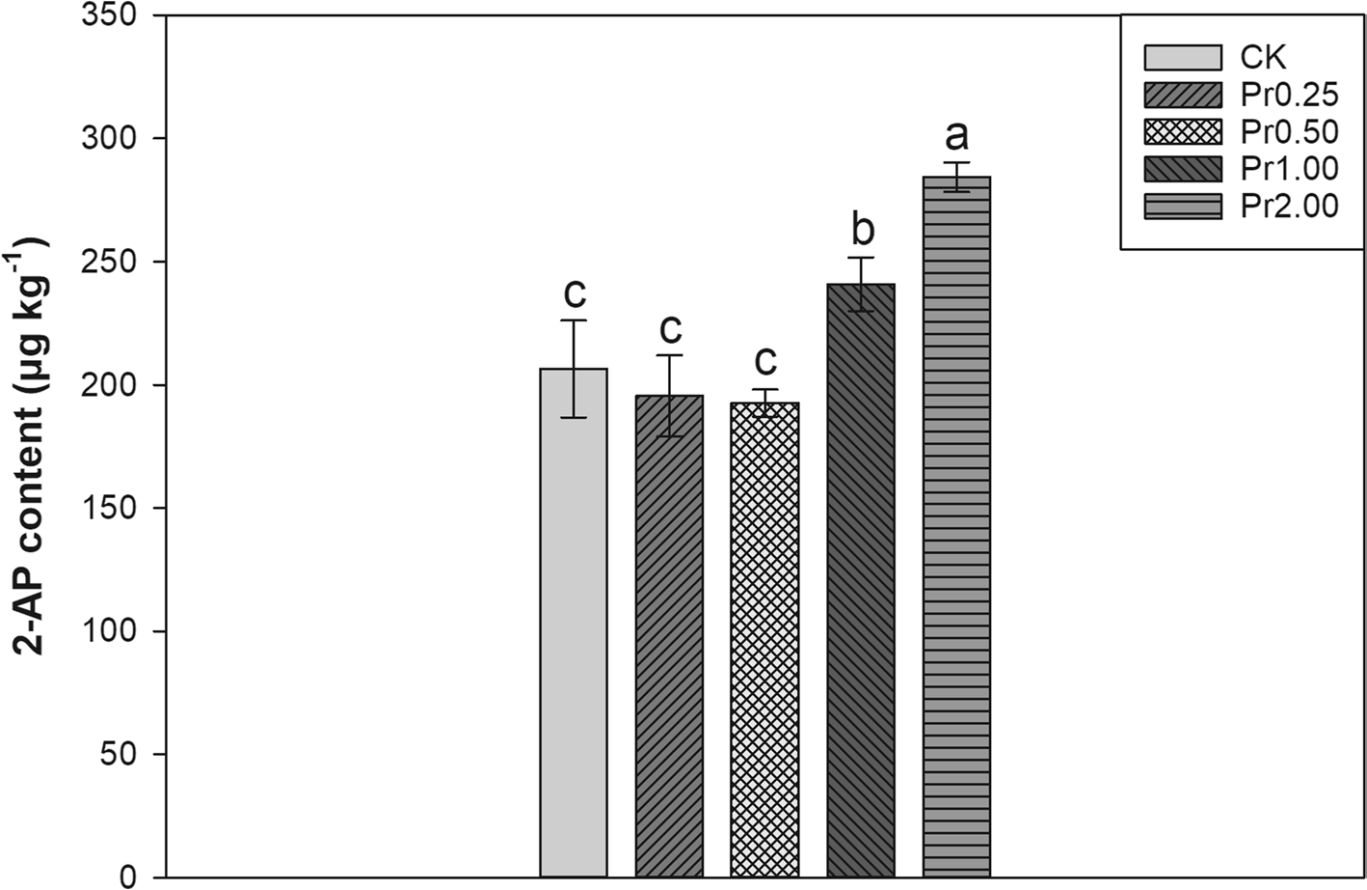
Effects of exogenous procyanidins on 2-AP content in aromatic rice. Values (means ± SEs) of each treatment were obtained from three independent replications (*n* = 3). Different letters indicate significant differences among the treatments (*P* < 0.05, least significant difference test)

Exogenous procyanidins substantially influenced the 2-AP content in aromatic rice (Fig. 1). Compared with CK, Pr1.00 and Pr2.00 treatments significantly increased 2-AP content by 16.67% and 37.68%, respectively. Pr0.25 and Pr0.50 treatments had no significant effect on 2-AP content compared with CK.

### Effects of exogenous procyanidins on proline, GABA, P5C, methylglyoxal, and 1-pyrroline contents

**Fig. 2:**
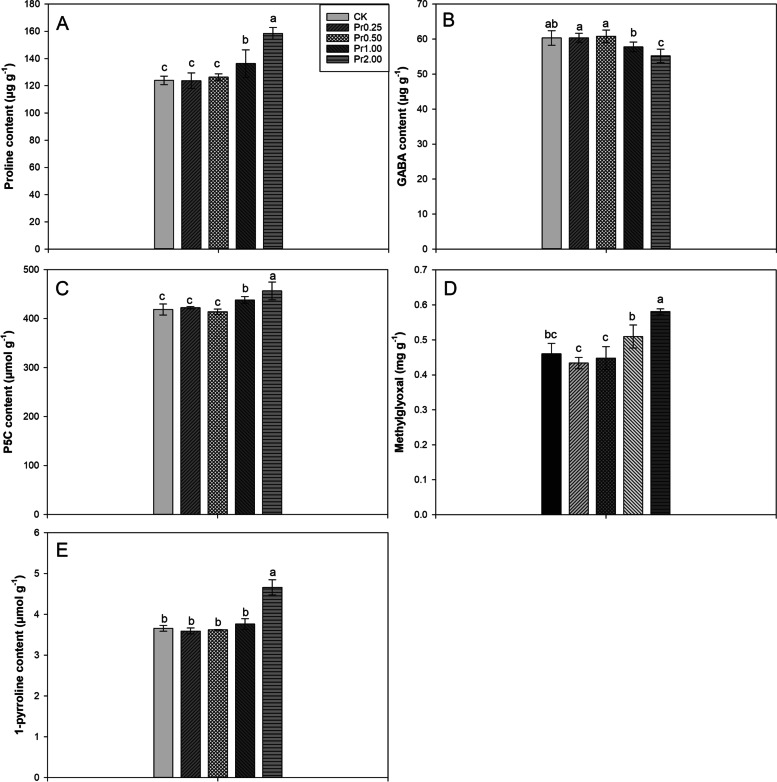
Effects of exogenous procyanidins on proline **A**, GABA (**B**), P5C (**C**), methylglyoxal (**D**), and 1-pyrroline (**E**) contents in aromatic rice. Values (means ± SEs) of each treatment were obtained from three independent replications (*n* = 3). Different letters indicate significant differences among the treatments (*P* < 0.05, least significant difference test)

As shown in Fig. 2, exogenous procyanidins substantially affected the contents of proline, GABA, P5C, methylglyoxal, and 1-pyrroline. Compared with CK, Pr1.00 and Pr2.00 treatments significantly increased proline content by 9.97% and 27.81%, respectively. 8.51% lower GABA content was recorded in Pr2.00 treatment than CK. 4.64% and 9.10% higher P5C contents were observed in Pr1.00 and Pr2.00 treatments than CK. Compared with CK, Pr1.00 and Pr2.00 treatments significantly increased methylglyoxal content by 10.74% and 26.63%, respectively. The content of 1-pyrroline in Pr2.00 treatment significantly increased by 27.46% compared with CK.

### Effects of exogenous procyanidins on genes related to 2-AP biosynthesis

**Fig. 3:**
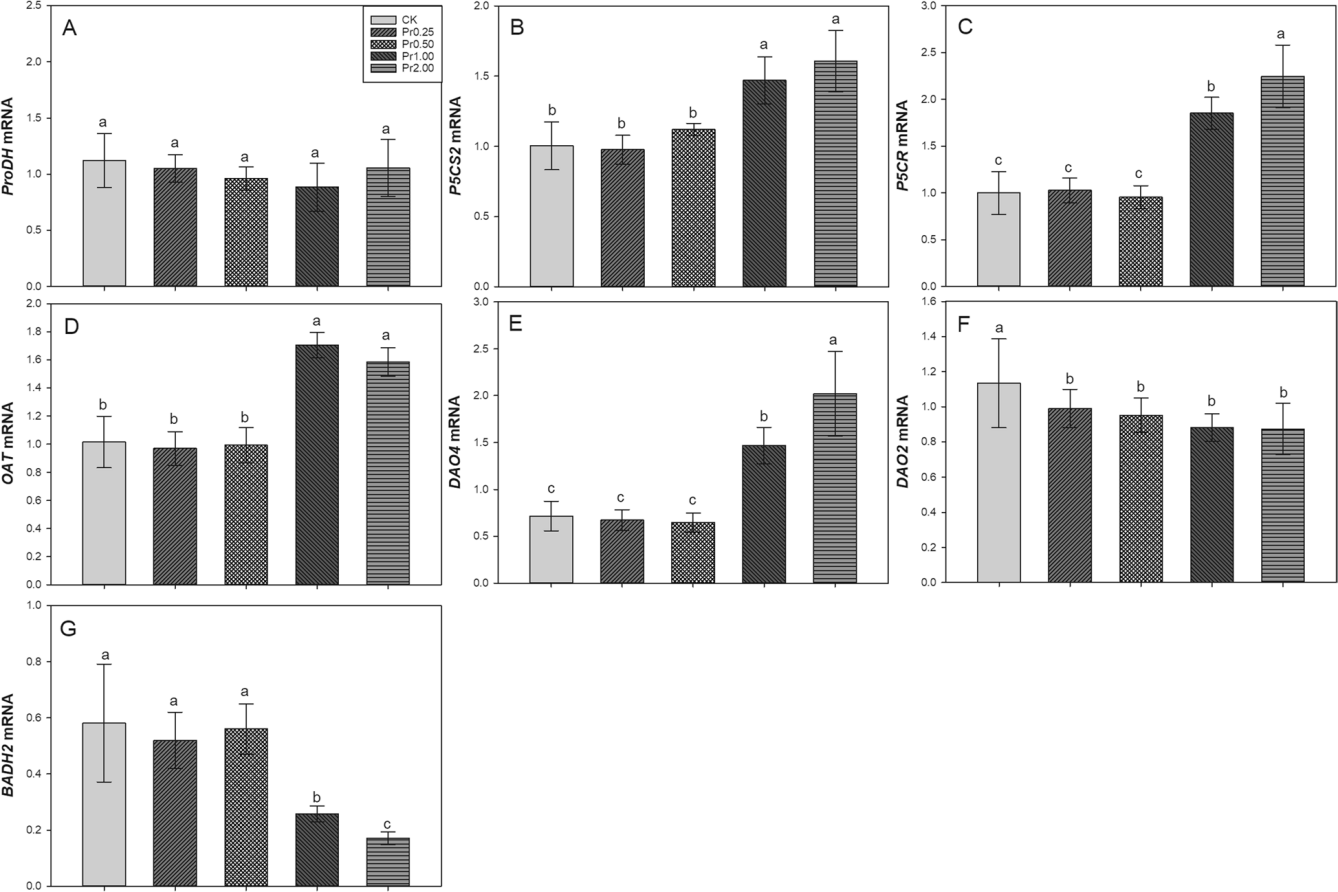
Effects of exogenous procyanidins on transcript levels of *ProDH* (**A**), *P5CS2* (**B**), *P5CR* (**C**), *OAT* (**D**), *DAO4* (**E**), *DAO2* (**F**), and *BADH2* (**G**) in aromatic rice. Values (means ± SEs) of each treatment were obtained from three independent replications (*n* = 9). Different letters indicate significant differences among the treatments (*P* < 0.05, least significant difference test)

Real-time PCR analysis showed that exogenous procyanidins substantially influenced the expression of some genes related to 2-AP biosynthesis in aromatic rice (Fig. 3). For *ProDH*, exogenous procyanidins had no significant effect on the transcript level of *ProDH*. For *P5CS2*, compared with CK, Pr1.00 and Pr2.00 treatments significantly up-regulated the transcript level of *P5CS2* by 46.39% and 60.10%, respectively. For *P5CR*, compared with CK, Pr1.00 and Pr2.00 treatments significantly up-regulated the transcript level of *P5CR* by 85.36% and 124.71%, respectively. For *OAT*, 67.83% and 56.03% higher transcript levels were recorded in Pr1.00 and Pr2.00 treatments than CK. For *DAO4*, compared with CK, Pr1.00 and Pr2.00 treatments significantly up-regulated the transcript level of *DAO4* by 104.82% and 181.86%, respectively. For *DAO2*, lower transcript levels were recorded in Pr0.25, Pr0.50, Pr1.00, and Pr2.00 treatments than in CK. There was no significant difference among the four procyanidins treatments. For *BADH2*, compared with CK, Pr1.00 and Pr2.00 treatments significantly down-regulated the transcript level of *BADH2* by 55.19% and 66.19%, respectively.

### Effects of exogenous procyanidins on PDH, P5CS, OAT, and BADH activities

**Fig. 4:**
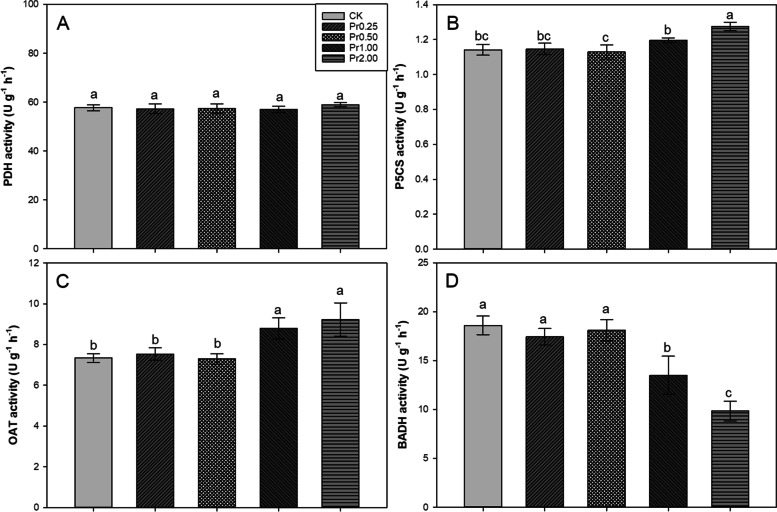
Effects of exogenous procyanidins on activities of PDH (**A**), P5CS (**B**), OAT (**C**), and BADH (**D**) in aromatic rice. Values (means ± SEs) of each treatment were obtained from three independent replications (*n* = 3). Different letters indicate significant differences among the treatments (*P* < 0.05, least significant difference test)

The activities of PDH, P5CS, OAT, and BADH were shown Fig. 4. Compared with CK, Pr2.00 treatment significantly enhanced P5CS activity by 11.65%. There was no significant difference among all treatments and CK in PDH activity. Compared with CK, Pr1.00 and Pr2.00 treatments significantly enhanced OAT activity by 9.62% and 25.84%, respectively. 55.76% and 70.58% lower BADH activities were recorded in Pr1.00 and Pr2.00 treatments compared with CK.

### Effects of exogenous procyanidins on growth parameters of aromatic rice plants

As shown in Table 1, exogenous procyanidins had no substantial effect on the growth of aromatic rice. There was no significant difference among all treatments and CK in plant height, stem diameter, fresh weight, and dry weight.

**Table 1:** Effect of exogenous procyanidins on the plant height, stem diameter, fresh weight, and dry weight of aromatic rice plants

Treatment	Plant height (cm)	Stem diameter (mm)	Fresh weight (mg)	Dry weight (mg)
CK	28.09 ± 0.78a	3.28 ± 0.22a	270.60 ± 8.77a	47.60 ± 0.58a
Pr0.25	28.36 ± 1.61a	3.28 ± 0.21a	258.00 ± 17.21a	48.38 ± 1.13a
Pr0.50	26.67 ± 0.38a	3.13 ± 0.06a	257.97 ± 11.81a	47.38 ± 0.35a
Pr1.00	27.04 ± 1.11a	3.27 ± 0.06a	267.77 ± 15.50a	46.93 ± 1.85a
Pr2.00	27.89 ± 0.40a	3.27 ± 0.13a	266.93 ± 26.74a	46.53 ± 0.48a

Values (means ± SEs) of each treatment were obtained from three independent replications (*n* = 9). Different letters indicate significant differences among the treatments (*P* < 0.05, least significant difference test)

## Discussion

2-AP is a key aromatic compound of the aroma of aromatic rice cultivars. The present study firstly reported an improvement of exogenous procyanidins on 2-AP biosynthesis in aromatic rice plants. The 2-AP content significantly increased under the foliar application of procyanidins, and the highest 2-AP content was recorded in Pr2.00 treatment. We observed that exogenous procyanidins significantly increased proline, 1-pyrroline, and P5C contents, which indicated that procyanidins enhanced 2-AP biosynthesis by increasing the contents of precursors. Our findings were supported by previous studies which showed that proline, 1-pyrroline, and P5C were important precursors in 2-AP formation [5, 22]. The study by Huang et al. [23] also revealed that the P5C formed from proline dehydrogenase catalyzed by PDH, was a precursor for 2-AP. In our study, the increment in proline content might be attributed to the up-regulated transcript levels of *P5CR* and *P5CS2* because a previous study indicated that glutamate was reduced to glutamate-semialdehyde by P5CS and spontaneously converted to P5C, and then P5CR further reduced the P5C intermediate to proline in plants [24]. Moreover, the study by Kayghobad et al. [7] showed that P5CS played a role in 2-AP biosynthesis, and overexpression of *P5CS2* would lead to a remarkable increase in 2-AP content in aromatic rice. Hence, we deduced that the improvement in transcript levels of *P5CR* and *P5CS2* played important roles in increased 2-AP under procyanidins treatment. Furthermore, we observed that exogenous procyanidins did not induce regulation in PDH activity or transcript level of *ProDH*, which indicated that procyanidins promoted 2-AP formation by enhancing proline biosynthesis rather than improving proline degradation. In addition, methylglyoxal content increased due to exogenous procyanidins, which also could be a reason for increased 2-AP because early studies revealed that 1-pyrroline would non-enzymatically react with methylglyoxal to produce 2AP in aromatic rice plants [25, 26]. In the present study, the improvement in methylglyoxal might be induced by increased 1-pyrroline content or other pathways, which need more studies to explore in the future.

In addition, we observed that procyanidins up-regulated the expression of *OAT* and enhanced the OAT activity. This finding indicated that procyanidins improved the ornithine metabolism to enhance 2-AP biosynthesis in aromatic rice. Our results were consistent with the study by Luo et al. [27] which showed that the conversion from ornithine to P5C was important to 2-AP production in aromatic rice, and foliar application of ornithine substantially increased 2-AP content. Moreover, there was no significant difference between Pr1.00 and Pr2.00 treatments in transcript level of *OAT* and OAT activity, which indicated the improvement of procyanidins in 2-AP biosynthesis through the ornithine pathway was limited.

**Fig. 5:**
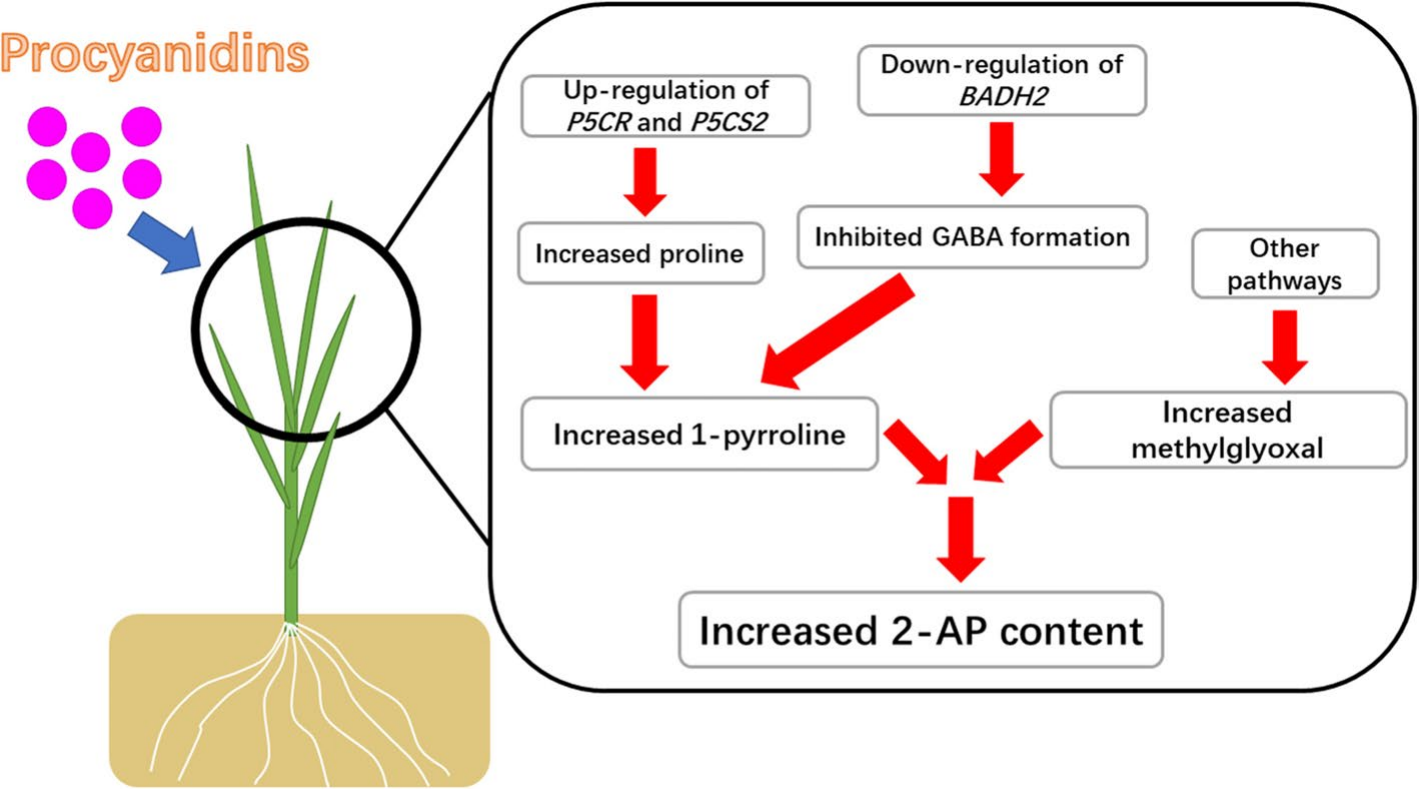
The possible regulation pathways of exogenous procyanidins on 2-AP biosynthesis in aromatic rice

Besides the proline pathway, there was another pathway to synthesize 2-AP in aromatic rice and this pathway was regulated by BADH and DAO [6, 11]. Previous studies described that DAO catalyzed putrescine to form γ-amino butyraldehyde which would cyclize to either Δ1-pyrroline or GABA, depending upon the functionality of *BADH2,* while the transformation from γ-amino butyraldehyde to GABA inhibited 2-AP production in aromatic rice [28]. Our results showed that exogenous procyanidins influenced this pathway through the up-regulation of the transcript level of DAO4 and the down-regulation of transcript levels of *DAO2* and *BADH2*. We also observed that exogenous procyanidins significantly reduced the GABA content. Therefore, we deduced that exogenous procyanidins not only enhanced proline biosynthesis to improve 2-AP production but also inhibited the transformation from γ-amino butyraldehyde to GABA to promote 2-AP biosynthesis. The possible regulation pathways of exogenous procyanidins on 2-AP were shown in Fig. 5. Overall, our study revealed the phenomenon that foliar application of procyanidins increased 2-AP content and preliminary explored the related and possible mechanism. However, our study had some limits. The biological function of these genes involved in procyanidins-mediated 2-acetyl-1-pyrroline biosynthesis remains largely unexplored. More deep research with mutants is needed in the future to fully reveal the mechanism behind the regulation of procyanidins on 2-AP in aromatic rice.

## Conclusion

Exogenous procyanidins significantly increased 2-AP content in aromatic rice plants. Higher proline, P5C, 1-pyrroline, and methylglyoxal contents and lower GABA content were observed due to exogenous procyanidins. Exogenous procyanidins also up-regulated the transcript levels of *P5CS2*, *P5CR*, *OAT*, *DAO4,* and down-regulated the transcript levels of *BADH2*. Enhanced activities of P5CS and OAT and diminished activity of BADH were recorded in procyanidins treatments. Our study firstly discovered the improvement of exogenous procyanidins on 2-AP biosynthesis in aromatic rice.

## Methods

### Experimental details and plant materials

The pot experiment was conducted with a well-known aromatic rice cultivar, *Xiangyaxiangzhan* (*Xiangsimiao126* × *Xiangyaruanzhan*), which was widely planted in South China, was provided by the College of Agriculture, South China Agricultural University, Guangzhou, China, in the College of Agriculture, South China Agricultural University, Guangzhou, China. Detailed information of the cultivar can be found at https://ricedata.cn/. This cultivar has been widely planted in South China for aromatic rice production and has been used many times for studies about aromatic rice [22, 29, 30]. After the germination, the seeds were sowed in a plastic pot (upper caliber 14.00 cm, lower caliber 9.20 cm, and height 8.30 cm) filled with paddy soil. Each pot was sowed with sixteen germinated seeds. The parameters of the chambers were set as 28/25 °C day/night temperature and 70% air relative humidity. During the growth of plants, the pots contained about a 1.00 cm water layer. Twenty days after the sowing, the aromatic rice plants were subjected to procyanidins treatments. Four procyanidins solutions with different concentrations (0.25 g L^−1^ (Pr0.25), 0.50 g L^−1^ (Pr0.50), 1.00 g L^−1^ (Pr1.00), and 2.00 g L^−1^ (Pr2.00)) were respectively sprayed to plants and ten pots for each treatment. In addition, the treatment sprayed with distilled water was taken as a control (CK). The procyanidins (CAS 485–22-6) was produced by Shanghai Yuanye Biotechnology Co., Ltd, Shanghai, China. Seven days after receiving the treatments, the plants were collected and stored at -80℃ for biochemical and molecular analysis. Meanwhile, the plant height, stem diameter, and fresh weight were measured. The dry weight was measured after being oven-dried at 60 °C for 48 h.

### Determination of 2-AP content

The 2-AP content was determined according to the methods of Okpala et al. [31] with a GCMS-QP 2010 Plus (Shimadzu Corporation, Japan). The samples were ground to powder with liquid nitrogen. Then, the 2-AP was extracted by adding dichloromethane and transferred to GC–MS machine for quantization. The final result was expressed as μg kg^−1^.

### Determination of proline, GABA, P5C, methylglyoxal, and 1-pyrroline contents

The proline content was determined according to Li et al. [32] with ninhydrin. The absorbance of the red chromophore in the toluene fraction was read at 520 nm, and proline content was expressed as μg g^−1^. GABA and P5C contents were determined according to the methods described by Bao et al. [22]. The GABA content was expressed as μg g^−1^, and P5C content was expressed as μmol g^−1^. The determination of methylglyoxal content was carried out according to Yadav et al. [33]. The reaction system consisted of 7.2 mM 1,2-diaminobenzene, 5 M perchloric acid, and the neutralized supernatant. The absorbance was read at 336 nm, and the final result was expressed as mg g^−1^. The determination of 1-pyrroline content was carried out according to Luo et al. [13]. The amount of 1- pyrroline in reaction mixtures containing 1,4-diaminobutane was determined immediately and after 30 min at room temperature. The absorbance was read at 430 nm, and the final result was expressed as μmol g^−1^.

### Determination of PDH, OAT, P5CS, and BADH activities

The determination of PDH activity was carried out according to the methods of Ncube et al. [34]. The absorbance was read at 440 nm after the reaction, and the activity was expressed as U g^−1^ min^−1^. The determination of OAT activity was carried out according to the methods of Luo et al. [11]. The reaction mixture consisted of potassium phosphate buffer (pH 8.0), pyridoxal-5-phosphate, ornithine, α-ketoglutarate, and the enzyme extract. The absorbance was read at 440 nm, and the final result was expressed as U g^−1^ min^−1^. The determination of P5CS was carried out according to the methods of Sanchez et al. [35]. The reaction mixture consisted of 50 mM L-glutamate, 20 mM magnesium chloride, 10 mM adenosine triphosphate, and 100 mM hydroxylamine hydrochloride, and the reaction was started by the addition of 0.5 ml of enzymatic extracts. The P5CS activity was expressed as U g^−1^ h^−1^. The determination of BADH activity was carried out according to the methods of Hibino et al. [36] and expressed as U g^−1^ min^−1^.

### Real-time quantitative RT-PCR

Real-time PCR analysis was carried out following the methods of Bao et al. [22]. Total RNA was extracted using the HiPure Plant RNA Mini Kit (Magen, Guangzhou, China). The quality and quantity of RNA were assessed by NanoDrop 2000. The Hiscript II QRT SuperMix for qPCR (+ gDNAwiper; Vazyme, Nanjing, China) synthesized cDNA from total RNA. Real-time quantitative RTPCR (qRT-PCR) was conducted in CFX96 real-time PCR System (Bio-Rad, Hercules, CA, USA). Each RNA sample was performed in triplicate. A negative control without cDNA template was always included. All primers were designed using Beacon Designer software (Premier Biosoft International, Palo Alto, CA). Primers are listed in Table 2.

**Table 2:** Primer sequences of genes encoding enzymes involved in 2-AP biosynthesis

Gene name	Accession No	Primer sequences
*Proline dehydrogenase (PRODH)*	AP014966.1	F 5'-TCATCAGACGAGCAGAGGAGAACAGG-3' R 5'-CCCAGCATTGCAGCCTTGAACC-3'
*Pyrroline-5-carboxylic acid synthetase2 (P5CS2)*	AP014957.1	F 5'-GAGGTTGGCATAAGCACAG-3' R 5'-CTCCCTTGTCGCCGTTC-3'
*Ornithine aminotransferase (OAT)*	AP014959.1	F 5'-GCCCTTGGTGCTGGAGTA-3' R 5'-AGCCCTTTCAACGAGACCTT-3'
*Diamine oxidase2 (DAO2)*	AP014960.1	F 5'-TCGTTCGCATCAAGGTTGG-3' R 5'-TCAGACAGAAGGGTGCCGTA-3'
*Diamine oxidase4 (DAO4)*	AP014960.1	F 5'-TGGCAAGATAGAAGCAGAAGT-3' R 5'-GTCCATACGGGCAACAAA-3'
*Betaine aldehyde dehydrogenase (BADH2)*	AB09683	F 5'-GGTTGGTCTTCCTTCAGGTGTGC-3' R 5'-CATCAACATCATCAAACACCACTAT-3'
*Pyrroline-5-carboxylate reductase (P5CR)*	AP014957.1	F 5'-TGGGCTAAGTGGTAGTGGC-3' R 5'-AGCTGACCCGGATGTTTT-3'

### Statistical analyses

The experimental data were analyzed with a one-way analysis of variance (ANOVA) using statistical software ‘Statistix 8.1′(Analytical Software, Tallahassee, FL, USA), while differences among means were separated using the least significant difference (LSD) test at the 5% probability level. The figures were presented using Sigma Plot 9.0 (Systat Software Inc., San Jose, CA, United States).

## Data Availability

All data generated or analysed during this study are included in this published article.

## Abbreviations

2-AP: 2-Acetyl-1-pyrroline
BADH: Betaine aldehyde dehydrogenase
DAO: Diamine oxidase
GABA: γ- Aminobutyric acid
OAT: Ornithine transaminase
PDH: Proline dehydrogenase
P5CS: 1-Pyrroline-5-carboxylate synthetase
P5C: 1-Pyrroline-5-carboxylic acid
P5CR: Pyrroline-5-carboxylate reductase

## References

[1] Dias LG, Hacke A, Dos Santos SE, Nath S, Canesin MR, Vilella OV, Geloneze B, Pallone JAL, Cazarin CBB, Blakeslee JJ (2022). Comparison of chemical and nutritional compositions between aromatic and non-aromatic rice from Brazil and effect of planting time on bioactive compounds. J Food Compos Anal.

[2] Jie Y, Shi T, Zhang Z, Yan Q (2021). Identification of Key Volatiles Differentiating Aromatic Rice Cultivars Using an Untargeted Metabolomics Approach. Metabolites.

[3] Zhao R, Luo H, Wang Z, Hu L (2020). Benefits of continuous plow tillage to fragrant rice performance. Agron J.

[4] Potcho PM, Okpala NE, Korohou T, Imran M, Kamara N, Zhang J, Aloryi KD, Tang X (2021). Nitrogen sources affected the biosynthesis of 2-acetyl-1-pyrroline, cooked rice elongation and amylose content in rice. PLoS ONE.

[5] Yoshihashi T, Huong NTT, Inatomi H (2002). Precursors of 2-Acetyl-1-pyrroline, a Potent Flavor Compound of an Aromatic Rice Variety. J Agr Food Chem.

[6] Chen S, Yang Y, Shi W, Ji Q, He F, Zhang Z, Cheng Z, Liu X, Xu M (2008). Badh2, Encoding Betaine Aldehyde Dehydrogenase, Inhibits the Biosynthesis of 2-Acetyl-1-Pyrroline, a Major Component in Rice Fragrance. Plant Cell.

[7] Kaikavoosi K, Kad TD, Zanan RL, Nadaf AB (2015). 2-Acetyl-1-Pyrroline Augmentation in Scented indica Rice (Oryza sativa L.) Varieties Through Δ1-Pyrroline-5-Carboxylate Synthetase (P5CS) Gene Transformation. Appl Biochem Biotech.

[8] Poonlaphdecha J, Gantet P, Maraval I, Sauvage F, Menut C, Morère A, Boulanger R, Wüst M, Gunata Z (2016). Biosynthesis of 2-acetyl-1-pyrroline in rice calli cultures: Demonstration of 1-pyrroline as a limiting substrate. Food Chem.

[9] Gay F, Maraval I, Roques S, Gunata Z, Boulanger R, Audebert A, Mestres C (2010). Effect of salinity on yield and 2-acetyl-1-pyrroline content in the grains of three fragrant rice cultivars (Oryza sativa L.) in Camargue (France). Field Crop Res.

[10] Kong L, Luo H, Mo Z, Pan S, Liu Z, Zhang Q, Bai S, Tang X (2020). Grain Yield, Quality and 2-Acetyl-1-Pyrroline of Fragrant Rice in Response to Different Planting Seasons in South China. Phyton-Int J Exp Bot.

[11] Luo H, Duan M, Kong L, He L, Chen Y, Wang Z, Tang X. The Regulatory Mechanism of 2-Acetyl-1-Pyrroline Biosynthesis in Fragrant Rice (Oryza sativa L.) Under Different Soil Moisture Contents. Front Plant Sci. 2021;12. 10.3389/fpls.2021.772728 PMC8660968 34899799

[12] Mo Z, Huang J, Xiao D, Ashraf U, Duan M, Pan S, Tian H, Xiao L, Zhong K, Tang X (2016). Supplementation of 2-Ap, Zn and La Improves 2-Acetyl-1-Pyrroline Concentrations in Detached Aromatic Rice Panicles In Vitro. PLoS ONE.

[13] Luo H, He L, Du B, Pan S, Mo Z, Duan M, Tian H, Tang X (2020). Biofortification with chelating selenium in fragrant rice: Effects on photosynthetic rates, aroma, grain quality and yield formation. Field Crop Res.

[14] Luo H, Du B, He L, Zheng A, Pan S, Tang X. Foliar application of sodium selenate induces regulation in yield formation, grain quality characters and 2-acetyl-1-pyrroline biosynthesis in fragrant rice. BMC Plant Biol. 2019;19(1). 10.1186/s12870-019-2104-4 PMC6858753 31730480

[15] Luo H, Duan M, He L, Yang S, Zou Y, Tang X (2021). A New Organic-Inorganic Compound Fertilizer for Improving Growth, Yield, and 2-Acetyl-1-Pyrroline Biosynthesis of Fragrant Rice. Agriculture (Basel).

[16] Mo Z, Li Y, Nie J, He L, Pan S, Duan M, et al. Nitrogen application and different water regimes at booting stage improved yield and 2-acetyl-1-pyrroline (2AP) formation in fragrant rice. Rice. 2019;12(1). 10.1186/s12284-019-0328-4 PMC6776583 31583492

[17] Rush MD, Rue EA, Wong A, Kowalski P, Glinski JA, van Breemen RB (2018). Rapid Determination of Procyanidins Using MALDI-ToF/ToF Mass Spectrometry. J Agr Food Chem.

[18] Oki T, Masuda M, Kobayashi M, Nishiba Y, Furuta S, Suda I, Sato T (2002). Polymeric procyanidins as radical-scavenging components in red-hulled rice. J Agr Food Chem.

[19] Valencia-Hernandez LJ, Wong-Paz JE, Ascacio-Valdés JA, Chávez-González ML, Contreras-Esquivel JC, Aguilar CN (2021). Procyanidins: From Agro-Industrial Waste to Food as Bioactive Molecules. Foods.

[20] Chen J, Chen Y, Zheng Y, Zhao J, Yu H, Zhu J, Li D. Neuroprotective Effects and Mechanisms of Procyanidins In Vitro and In Vivo. Molecules. 2021;26(10). 10.3390/molecules26102963 PMC8155916 34067571

[21] Fang L, Li M, Zhao L, Han S, Li Y, Xiong B, Jiang L (2020). Dietary grape seed procyanidins suppressed weaning stress by improving antioxidant enzyme activity and mRNA expression in weanling piglets. J Anim Physiol An N.

[22] Bao G, Ashraf U, Wang C, He L, Wei X, Zheng A, Mo Z, Tang X (2018). Molecular basis for increased 2-acetyl-1-pyrroline contents under alternate wetting and drying (AWD) conditions in fragrant rice. Plant Physiol Bioch.

[23] Huang T, Huang Y, Hung H, Ho C, Wu M. δ1-Pyrroline-5-carboxylic Acid Formed by Proline Dehydrogenase from theBacillus subtilis ssp. natto Expressed inEscherichia coli as a Precursor for 2-Acetyl-1-pyrroline. J Agr Food Chem. 2007;55(13):5097-5102. 10.1021/jf0700576 17536821

[24] Szabados L, Savouré A (2010). Proline: a multifunctional amino acid. Trends Plant Sci.

[25] Wakte K, Zanan R, Hinge V, Khandagale K, Nadaf A, Henry R (2017). Thirty-three years of 2-acetyl-1-pyrroline, a principal basmati aroma compound in scented rice (Oryza sativa L.): a status review. J Sci Food Agr.

[26] Bradbury LMT, Gillies SA, Brushett DJ, Waters DLE, Henry RJ (2008). Inactivation of an aminoaldehyde dehydrogenase is responsible for fragrance in rice. Plant Mol Biol.

[27] Luo HW, Xing PP, Liu JH, Lai RF, He LX, Zhang TT, Tang XR (2020). Application of ornithine-induced regulation in yield formation, grain quality and aroma of fragrant rice. Cereal Res Commun.

[28] Ghosh P, Roychoudhury A: Differential levels of metabolites and enzymes related to aroma formation in aromatic indica rice varieties: comparison with non-aromatic varieties. 3 Biotech 2018, 8(1). 10.1007/s13205-017-1045-6 PMC5736499 29279818

[29] Bao G, Ashraf U, Wan X, Zhou Q, Li S, Wang C, He L, Tang X (2021). Transcriptomic Analysis Provides Insights into Foliar Zinc Application Induced Upregulation in 2-Acetyl-1-pyrroline and Related Transcriptional Regulatory Mechanism in Fragrant Rice. J Agr Food Chem.

[30] Liu X, Huang Z, Li Y, Xie W, Li W, Tang X, Ashraf U, Kong L, Wu L, Wang S, et al. Selenium-silicon (Se-Si) induced modulations in physio-biochemical responses, grain yield, quality, aroma formation and lodging in fragrant rice. Ecotox Environ Safe. 2020;196:110525. 10.1016/j.ecoenv.2020.110525 32224370

[31] Okpala NE, Potcho MP, An T, Ahator SD, Duan L, Tang X (2020). Low temperature increased the biosynthesis of 2-AP, cooked rice elongation percentage and amylose content percentage in rice. J Cereal Sci.

[32] Li YH, Tian P, Li CZ, Yu XZ: Elucidating comportment of the glutamate and ornithine pathway on proline accumulation in rice under different nitrogenous nutrition. INT J Environ Sci Te. 2021;19(4):2993–3000.

[33] Yadav SK, Singla-Pareek SL, Ray M, Reddy MK, Sopory SK (2005). Methylglyoxal levels in plants under salinity stress are dependent on glyoxalase I and glutathione. Biochem Bioph Res Co.

[34] Ncube B, Finnie JF, Van Staden J (2013). Dissecting the stress metabolic alterations in in vitro Cyrtanthus regenerants. PLANT PHYSIOL BIOCH.

[35] Sánchez E, Ruiz JM, Romero L (2002). Proline metabolism in response to nitrogen toxicity in fruit of French Bean plants (Phaseolus vulgaris L. cv Strike). Sci Hortic-Amsterdam.

[36] Hibino T, Meng YL, Kawamitsu Y, Uehara N, Matsuda N, Tanaka Y, Ishikawa H, Baba S, Takabe T, Wada K (2001). Molecular cloning and functional characterization of two kinds of betaine-aldehyde dehydrogenase in betaine-accumulating mangrove Avicennia marina (Forsk.) Vierh. Plant Mol Biol.

